# piRNA clusters and open chromatin structure

**DOI:** 10.1186/1759-8753-5-22

**Published:** 2014-08-01

**Authors:** Soichiro Yamanaka, Mikiko C Siomi, Haruhiko Siomi

**Affiliations:** 1 Department of Molecular Biology, Keio University School of Medicine, 35 Shinanomachi Shinjuku-ku, Tokyo 160-8582, Japan; 2 Department of Biological Sciences, Graduate School of Science, The University of Tokyo, Tokyo 113-0032, Japan

**Keywords:** Transposable elements, Piwi, piRNA, piRNA cluster, Chromatin boundary

## Abstract

Transposable elements (TEs) are major structural components of eukaryotic genomes; however, mobilization of TEs generally has negative effects on the host genome. To counteract this threat, host cells have evolved genetic and epigenetic mechanisms that keep TEs silenced. One such mechanism involves the Piwi-piRNA complex, which represses TEs in animal gonads either by cleaving TE transcripts in the cytoplasm or by directing specific chromatin modifications at TE loci in the nucleus. Most Piwi-interacting RNAs (piRNAs) are derived from genomic piRNA clusters. There has been remarkable progress in our understanding of the mechanisms underlying piRNA biogenesis. However, little is known about how a specific locus in the genome is converted into a piRNA-producing site. In this review, we will discuss a possible link between chromatin boundaries and piRNA cluster formation.

## Review

### Background

Large fractions of eukaryotic genomes comprise transposable elements (TEs), which are repetitive DNA elements that can mobilize to take up new chromosomal locations within a genome. TEs act as insertional mutagens that can alter gene expression or rearrange chromosomes. Therefore, they can cause disease and may even drive evolution [1-4]. TEs are diverse in sequence and in the way they transpose [5,6]. They possess a limited gene set of their own, but use the gene expression machinery of their host to thrive in the genome. DNA transposons move by a “cut-and-paste” mechanism, in which they are excised from one genomic site and inserted into a new location using their own transposase. Therefore, generally, the copy number of DNA transposons in a genome does not expand. By contrast, retrotransposons use a “copy-and-paste” mechanism to propagate their copies through RNA intermediates. Retrotransposons are transcribed from the genome, reverse transcribed and integrated into a new location, in a process mediated by a transposon-encoded reverse transcriptase. Retrotransposons are distinguished by their DNA sequence topology and mechanism of transposition: those that possess long terminal repeats (LTRs), such as *gypsy*, and those that do not (non-LTRs), such as long interspersed repetitive elements (LINEs) and short interspersed repetitive elements (SINEs). Both DNA transposons and retrotransposons have non-autonomous subtypes and defective copies, which require the reverse transcriptase and endonuclease supplied by the autonomous type to jump around the genome.

As an example, *Drosophila* harbors around 100 different TEs, and the only conserved and universal property shared by them is the ability of transposition [7]. Thus, the requirements for host cells for repression of TEs are at least two-fold: 1) a mechanism that recognizes such a diverse set of TE types, and 2) a mechanism that distinguishes them from other cellular genes and selectively targets them for silencing. Recent studies have postulated that host cells have evolved an elaborate silencing mechanism to meet these two requirements. Host cells may have taken advantage of the only universal property of TEs, their transposition ability to trap them in specific genomic locations and subject them to a silencing program, which employs small RNA-based immunity to selectively silence homologous elements [8-10]. In animal gonads, small non-coding RNAs (ncRNAs), termed Piwi-interacting RNAs (piRNAs), mediate TE silencing to ensure genome integrity during germ cell development [11,12]. Most piRNAs are derived from particular genomic sites termed piRNA clusters, which contain a large number and various types of TEs. Thus, the sequences of piRNAs derived from these clusters are homologous not only to TEs in the clusters, but also to related TEs located elsewhere in the genome and can therefore act as guide molecules to repress TEs *in trans*. Thus, piRNA clusters are genetic elements that regulate the activity of TEs. However, relatively little is known about how piRNA clusters are formed. In this review, we emphasize the role of chromatin boundaries in piRNA cluster formation. To this end, we briefly review our current knowledge of piRNAs and piRNA clusters. We then discuss a possible link between chromatin boundaries and piRNA clusters, and propose some models as to how piRNA clusters are formed in chromatin boundaries.

### TE silencing mediated by piRNAs

RNA interference (RNAi) and related pathways are cellular pathways in which small ncRNAs of 20 to 35 nucleotides (nt) guide Argonaute-containing effector complexes, termed RNA-induced silencing complexes (RISCs), to RNA targets by means of base-pairing, and promote the inactivation of homologous sequences [13-16]. They have been shown to suppress the activity of TEs in plants and animals. In animal germline cells, piRNAs of 24 to 35 nt are produced and loaded onto germline-specific Argonaute proteins (termed PIWI proteins) to form piRNA-induced silencing complexes (piRISCs). Genetic analyses of *Drosophila PIWI* genes (*ago3*, *aubergine/aub*, and *piwi*) have revealed that mutations in these genes affect germline development [17-20]. TEs are deregulated in mutant ovaries defective in these genes, suggesting a model in which TE overexpression and mobilization triggers DNA damage signaling-dependent defects in an early step in the germ cell patterning cascade [21].

**Figure 1:**
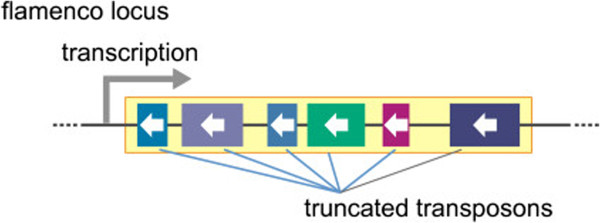
***flamenco*, a major Piwi-interacting RNA (piRNA) cluster in somatic support cells of the *Drosophila *ovary.** The *flamenco* locus contains a particular family of transposon (boxes with white arrows; the arrows denotes the direction of each transposon) in its transcription unit. Almost all transposons are truncated and/or inactivated. The direction of the transposons is exclusively anti-sense with regard to transcription in this region (gray arrow). This region spans about 150 kb, and is thought to behave as a single transcriptional unit.

**Figure 2:**
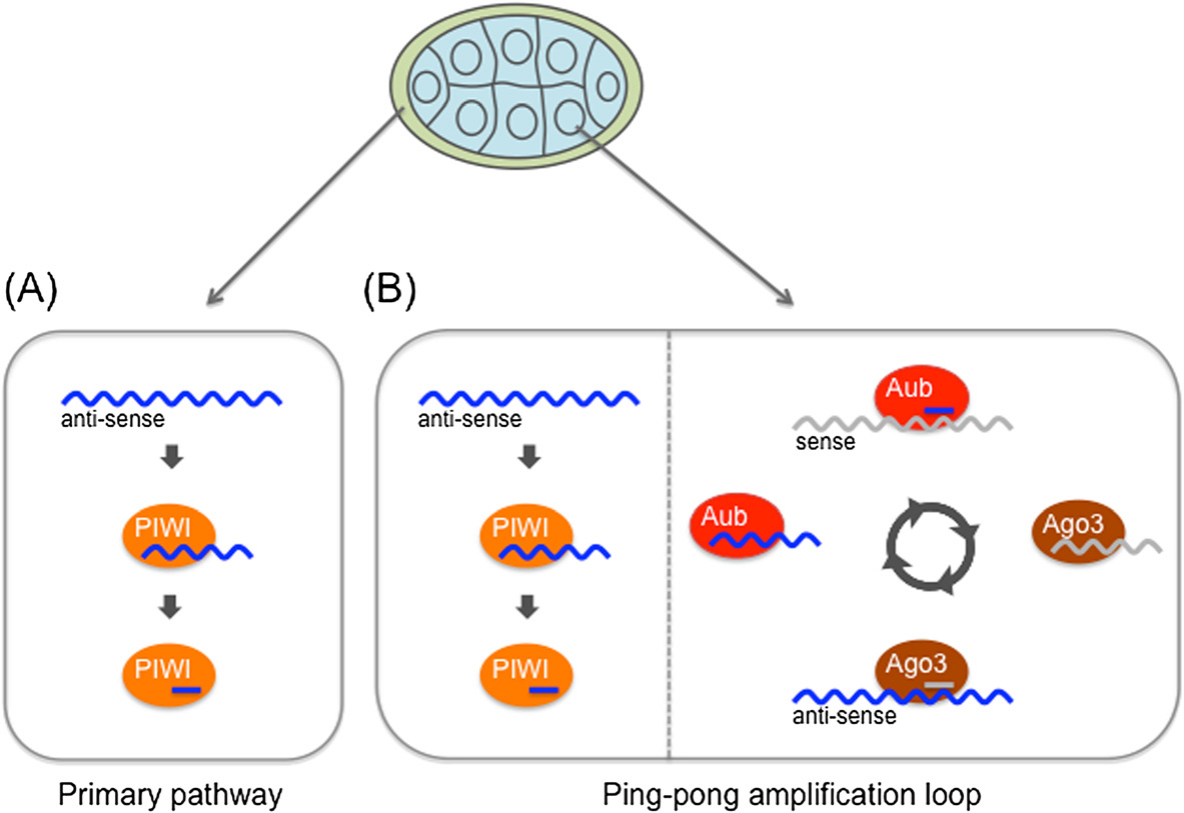
**Piwi-interacting RNA (piRNA) biogenesis pathway in the *Drosophila *ovary. (A)** Primary piRNA pathway in somatic support cells (cream region surrounding the central egg). The transposon sequence in piRNA clusters (the majority are unistrand clusters; see Figure 5 below) in somatic support cells is in an exclusively anti-sense orientation with regard to the direction of transcription. The resultant transcripts are transported to the cytoplasm, recognized, and processed by several factors, including Zucchini, Armi, and Yb. Finally, they are loaded onto the PIWI protein. **(B)** The ping-pong amplification loop in germ cells (light blue region). Transcripts from piRNA clusters (mainly dual-strand clusters; see Figure 5 below) and active transposons are processed into piRNAs by Aub and Ago3. piRNAs from sense transposon transcript are preferentially loaded onto Ago3, and those from anti-sense transposon transcript are preferentially loaded onto Aub.

Unlike other small silencing RNAs such as microRNAs (miRNAs) and small interfering RNAs (siRNAs), piRNAs in most animals are processed in a Dicer-independent manner from single-stranded precursors, which are transcribed mostly from genomic piRNA clusters [22,23]. A large number of genes have been identified to function in piRNA biogenesis [24]. In the *Drosophila* genome, 142 regions have been identified as piRNA clusters [22]. Although these sites account for less than 5% of the assembled genome, over 90% of all sequenced piRNAs can be derived from these regions [25]. The piRNA clusters cover chromosomal regions of several to hundreds of kilobases, and they contain TEs that are mostly inactive copies or truncated fragments, arranged in a nested fashion [22]. Among all the piRNA clusters in *Drosophila*, the *flamenco* locus produces a major fraction of piRNAs in somatic support cells in the ovary [25]. This locus was originally discovered as a regulator of the activity of the *gypsy*, *idefix*, and *ZAM* TEs [26,27]. piRNAs from this cluster, which spans about 150 kb, are derived from one DNA strand only, most likely through unidirectional transcription oriented in the anti-sense direction to most TEs in the locus (Figure 1). This provides a molecular basis of why Piwi, the only PIWI protein expressed in ovarian somatic cells, loads with piRNAs that are anti-sense-oriented relative to TEs. Mutants of *flamenco* in which the *P-element* is inserted in the 5′ region and those lacking *flamenco* partial genomic sequence lose the ability to regulate TEs [22,26,28,29]. These data indicate that the single long transcripts from the *flamenco* locus are processed into piRNAs. This linear biogenesis of piRNAs from precursor transcripts has been called the ‘primary piRNA processing pathway’ (Figure 2). piRNA maturation and Piwi-piRNA complex (Piwi-piRISC) formation occur in the cytoplasm [30]. Piwi-piRISCs are then imported into the nucleus where they repress TEs *in trans* at transcriptional level by directing specific histone modifications to TE loci [31-34]. This suggests that Piwi-piRISCs recruit the relevant enzymes to modify histones at TE loci. Because depletion of *piwi* activity rapidly results in derepression of TEs, the TE silencing state requires the continual activities of Piwi-piRISCs [30,35,36]. Therefore, Piwi-piRISCs are genetic elements that mediate and maintain epigenetic chromatin modifications of target TE loci.

Compared with the situation in somatic support cells, the piRNA biogenesis in germline cells in the fly ovary is more complex. In contrast to the unidirectional *flamenco* piRNA cluster, many piRNA clusters in the *Drosophila* germline are transcribed from both strands, and both precursor transcripts are processed into piRNAs [22,25]. Therefore, both sense and anti-sense piRNAs relative to the TE sequences are produced from the clusters. All three PIWI proteins are expressed in the germline, but Piwi is nuclear, and both Aub and Ago3 are cytoplasmic [22,37,38]. Anti-sense precursor transcripts from dual-stranded piRNA clusters are processed into anti-sense piRNAs that are loaded onto Aub and Piwi (“primary piRNA processing pathway”). Piwi-piRISCs then move into the nucleus where they repress TEs, probably by a mechanism similar to that observed in somatic support cells. Aub-piRISCs, by contrast, remain in the cytoplasm and cleave both sense precursor transcripts from dual-stranded piRNA clusters and transcripts from active TEs, using the small RNA-directed endonuclease or Slicer activity exhibited by PIWI proteins [37]. This cleavage results in the production of sense piRNAs, which in turn are loaded onto Ago3. This process initiates a feed-forward amplification loop of piRNA production, the so-called “ping-pong cycle”, in which sense and anti-sense transcripts of dual-stranded piRNA clusters and active TEs are reciprocally cleaved by the Slicer activity of Ago3 and Aub [22,37] (Figure 2). Both Ago3-piRISCs and Aub-piRISCs act catalytically, and thus the cycle leads to repeated rounds of piRNA production by consuming both cluster transcripts and TE transcripts, thereby silencing TEs at posttranscriptional levels in the cytoplasm.

**Figure 3:**
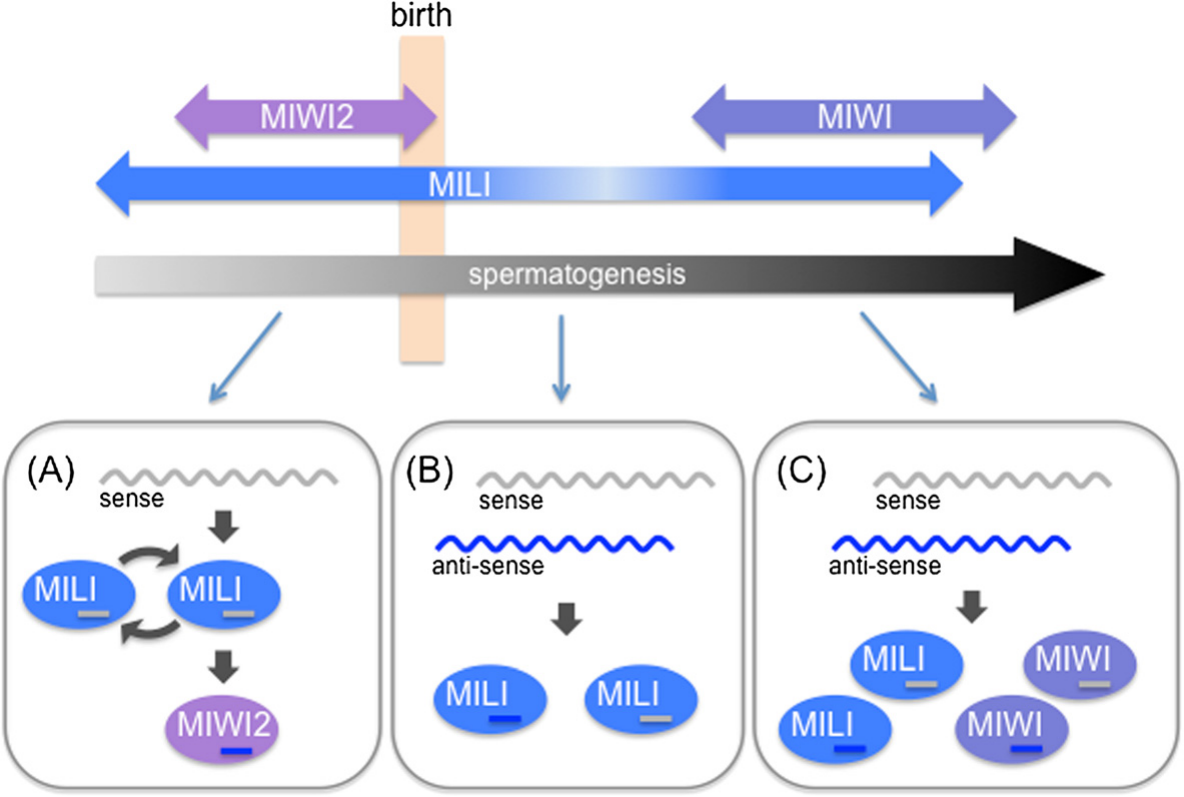
**The Piwi-interacting RNA (piRNA) biogenesis pathway in mouse testis.** The piRNA biogenesis pathway in mouse can be categorized into three modes. MILI is expressed in both prenatal and adult testis. MIWI2 is expressed in prenatal testis and its expression decreased after birth and is not detectable in adult testis. MIWI is expressed in adult testis. **(A)** When MILI and MIWI2 are coexpressed in prenatal testis, the primary piRNA transcript is processed for loading into MILI. The MILI-piRISC can form homotypic ping-pong amplification loop. MIWI2-associated piRNAs are processed from anti-sense transcripts with the aid of MILI-piRNA-induced silencing complex (piRISC). Therefore, the production of MIWI2-associated piRNA depends on mature MILI-piRISC. **(B)** When only MILI protein is expressed in testis, MILI process sense and anti-sense piRNA precursor transcripts. **(C)** When MILI and MIWI are coexpressed in adult testis, both Piwi proteins process the sense and anti-sense piRNA precursor transcript.

The mouse genome encodes three distinct PIWI proteins: MIWI, MIWI2, and MILI. In contrast to *Drosophila* PIWI proteins, which are expressed in both male and female gonads, the expression of mouse PIWI proteins is rather restricted to male gonads [39-41]. Male knock-out (KO) mice for each *PIWI* gene show defects in spermatogenesis and sterility, but female *PIWI* KO mice are normal [39-41]. Two distinct piRNA populations are present in mouse testes: the pre-pachytene and pachytene piRNA pools. Pre-pachytene piRNAs are enriched in TE-derived sequences (approximately 80% of the total), and associate with MIWI2 and MILI [39]. Pachytene piRNAs, by contrast, have a higher proportion of unannotated sequences, with diminished contribution from TE-derived sequences (approximately 25%) [42-44]. Pachytene piRNAs enter MILI and MIWI [42-45] (Figure 3). Similar to the case in *Drosophila*, both the primary piRNA processing pathway and the ping-pong cycle operate in mouse testes. MILI and MIWI accommodate piRNAs from the primary piRNA processing pathway, but unlike in *Drosophila*, mouse primary piRNAs are predominantly sense-oriented relative to the TE transcripts [11]. It was initially thought that MILI and MIWI2 form a ping-pong amplification loop, and that anti-sense piRNAs were loaded onto MIWI2 to form MIWI2-piRISCs [39,46]. However, recent studies have shown that the Slicer activity of MILI is required for the secondary piRNA production, which amplifies MILI-bound piRNAs through an intra-MILI ping-pong loop, and generates all MIWI2-bound secondary piRNAs [45] (Figure 3). In contrast to the cytoplasmic localization of MILI and MIWI, MIWI2-piRISCs are imported into the nucleus where they direct specific DNA methylation of TE loci, thereby establishing TE silencing at the transcriptional level [39,45,47]. However, the Slicer activity of both MIWI and MILI is still required to maintain TE silencing in the mouse testis after birth, suggesting that continuous cleavage of TE transcripts by the Slicer activity is essential to repress TEs in mouse testes [44,45].

### piRNA clusters in diverse organisms

**Figure 4:**
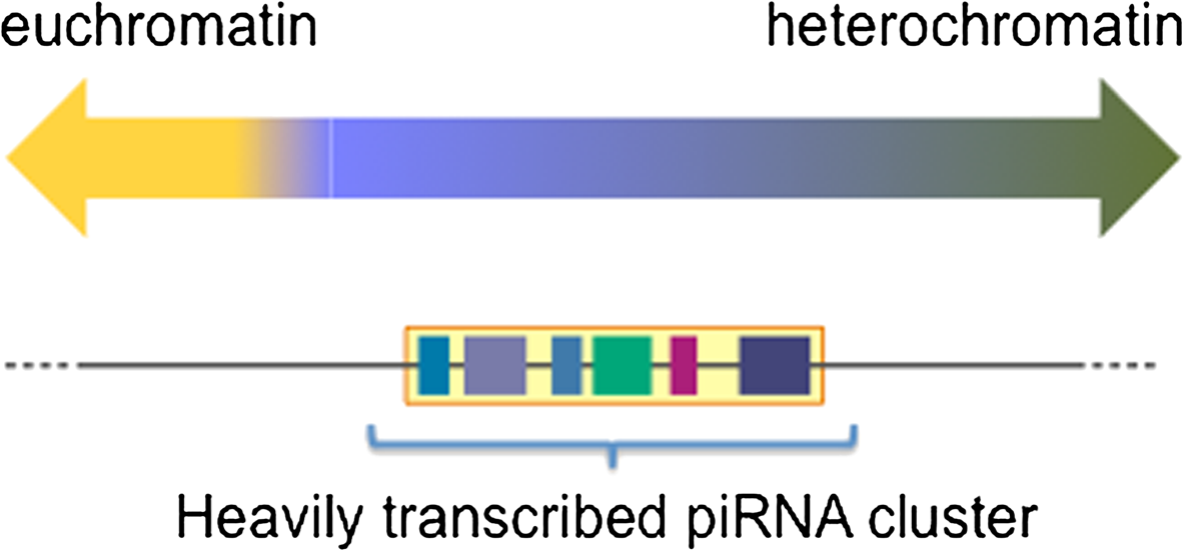
**Most *Drosophila *Piwi-interacting RNA (piRNA) clusters are found near the boundary zone between euchromatin and heterochromatin.** The boundary between euchromatin and heterochromatin of *Drosophila* is gradual rather than acute. Most *Drosophila* piRNA clusters exist in the boundary zone between euchromatin and heterochromatin.

TE insertions in *Drosophila* are mostly located in heterochromatin and proximal heterochromatin-euchromatin boundary zones [22]. Of 142 piRNA clusters identified in *Drosophila*, only 7 are in presumed euchromatic regions, while the rest reside within cytologically defined pericentromeric and telomeric heterochromatin regions. Within these heterochromatin regions, the piRNA clusters tend to be located near the boundary region between heterochromatin and euchromatin. Heterochromatin regions in the *Drosophila* genome can be found at the pericentromeric and subtelomeric regions, and are megabases in size [48-50]. Their constituent sequences fall into roughly three categories: tandemly repeated short sequences (satellite DNAs), moderately repetitive elements (such as TEs), and some single-copy genes [48-50]. In the *Drosophila* genome, intact and potentially active TEs prevail across the genome, while fragmented or inactive copies of TEs are strongly enriched in the transition zones between heterochromatin and euchromatin near to the centromere, and constitute piRNA clusters [22,50] (Figure 4).

Because most piRNAs are derived from piRNA clusters that genetically control the activity of TEs and largely comprise various types of defective TEs, a model in which piRNA clusters act as “TE traps” has been proposed [8,51-53]. This model relies on the transposition ability of TEs for piRNA clusters to passively acquire new content by chance transposition. TEs that happen to jump into piRNA clusters can then become fixed by evolutionary selection, and produce corresponding piRNAs and regulate other homologous elements expressed from different genomic positions in germ cells.

**Figure 5:**
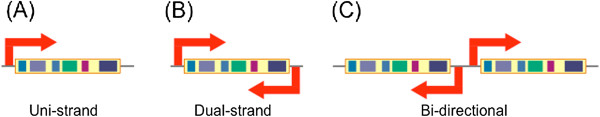
**Three types of Piwi-interacting RNA (piRNA) cluster. (A)** Unistrand piRNA cluster; piRNAs are produced from only one genomic DNA strand. **(B)** Dual-strand piRNA cluster; piRNAs are produced from both strands of the same genomic region. **(C)** Bidirectional piRNA cluster; two unistrand piRNA clusters are arranged in a divergent manner.

As mentioned above, two types of piRNA clusters exist in the *Drosophila* gonads: unidirectional clusters and dual-stranded clusters. Most piRNA clusters in somatic support cells are unidirectional, while the predominant fraction of germline piRNA clusters is dual-stranded [22,25] (Figure 5).

An example of a unidirectional piRNA cluster is the *flamenco* locus, which is located near the pericentromeric heterochromatin boundary of the X chromosome, and contains a large number of truncated or inactivated TEs. Most of these TEs belong to the *gypsy* family and are anti-sense-oriented with regard to the polarity of transcription. This requires the transcription factor Cubitus interruptus, a segment polarity gene that controls a number of genes, including Hedgehog genes [22,54]. The molecular mechanism that restricts the directionality of transposition into a unistrand piRNA cluster is not well understood.

A representative dual-stranded cluster is the 42AB cluster, which spans around 240 kb, near the pericentromeric heterochromatin boundary. However, the orientation of truncated TEs in this cluster is random rather than uniform, and piRNAs are produced from both sense and anti-sense strands.

Although many factors that are required for piRNA production are shared between these two types of clusters, there are some differences between them. Rhino (a variant of heterochromatin protein 1; HP1), Cutoff (a homolog of the yeast decapping nuclease and transcription termination factor Rai1), and Deadlock (which acts as a linker between Rhino and Cutoff), are all required for piRNA production only in germline cells of the oocyte [22,55-57]. Interestingly, most piRNA clusters in *Drosophila* are within cytologically defined heterochromatic regions. A recent chromatin immunoprecipitation (ChIP)-sequencing analysis of H3K9me3, the most established marker for heterochromatic regions, revealed that the promoter and its surrounding region of *flamenco*, a unistrand piRNA cluster, is fairly devoid of this repressive histone mark, which may explain the active transcription of the locus by RNA polymerase II [34]. By contrast, the germline cell-specific dual-strand piRNA clusters, such as 42AB, are coated with H3K9me3, but are still transcriptionally active [55] (see also below).

In the *Bombyx mori* tissue cultured cell line BmN4, a portion of piRNAs are derived from TEs [58]. piRNA clusters in BmN4 cells have been shown to have a high level of H3K4me3 mark, which is a hallmark of active transcription [59], suggesting the open nature of silkworm piRNA clusters.

These findings suggest that piRNA clusters are highly transcribed units within heterochromatic regions, and raise the question of how this kind of special location in the genome has been selected for piRNA clusters to produce piRNAs.

In the mouse, over 90% of piRNA reads have been mapped to roughly 100 genomic regions, ranging from a few kb to over 100 kb in length. Most mouse clusters show profound strand asymmetry, with reads arising from only one strand within a cluster (unidirectional cluster). When piRNAs map to both strands within one piRNA cluster, the transcription units are arranged in a divergent manner (bidirectional cluster) [42,43] and the piRNA-producing region on one strand does not overlap with that on the other strand. In prenatal mouse testes, piRNAs are produced from both strands in the same region (dual-strand cluster) [39] (Figure 5). Recent comprehensive deep sequencing analysis of postnatal mouse testes reveals that the transcription factor A-MYB drives pachytene piRNA production, suggesting a model in which a specific transcription factor engages in transcription of most piRNA clusters [60,61]. It should be noted that A-MYB is not specific for piRNA clusters, but rather has a number of target genes, suggesting that A-MYB has been co-opted to drive transcription of piRNA clusters. This also raises the question of what might be the difference between the A-MYB binding sites that direct piRNA production and the A-MYB binding sites that produce mRNAs but not piRNAs. piRNA clusters have been identified in other mammals including primates [62]. Synteny analysis has revealed conservation in the genomic location of piRNA clusters among mammals, although the precise sequence of each piRNA shows no apparent similarity [42,43,62]. This indicates that the relative chromosomal position has some marked features with regard to production of piRNAs, and such special features are maintained across mammals.

*Caenorhabditis elegans* has two PIWI proteins, PRG-1 and PRG-2. PRG-1 is required in germline maintenance, and interacts with a class of small RNAs, called 21U-RNAs [63,64]. By definition, 21U-RNAs are the piRNAs of *C. elegans*. As their name implies, they are characterized by a first U bias, and their length is exclusively 21 nt, which is shorter than that of piRNA species in other organisms [65]. The vast majority of the 21U-RNAs are derived from thousands of individual loci broadly scattered in two large clusters on chromosome IV [65]. These regions are gene-poor compared with other regions of the genome. A marked feature of 21U-RNAs is the existence of a clear *cis* motif located around 40 bp upstream of the 21U-RNA encoding site [65]. The consensus motif is CTGTTTCA and is flanked by an AT-rich sequence, which is specifically recognized by Forkhead family transcription factors [65,66]. In addition, ChIP-on-chip experiments have shown a low level of histone H3 across the two piRNA clusters, which correlates well with DNase-sensitive sites [66,67]. Moreover, it was also revealed that each upstream consensus motif corresponds with the nucleosome-depleted region (NDR) [66]. These findings strongly suggest that each piRNA in *C. elegans* is produced from an independent transcription unit.

**Figure 6:**
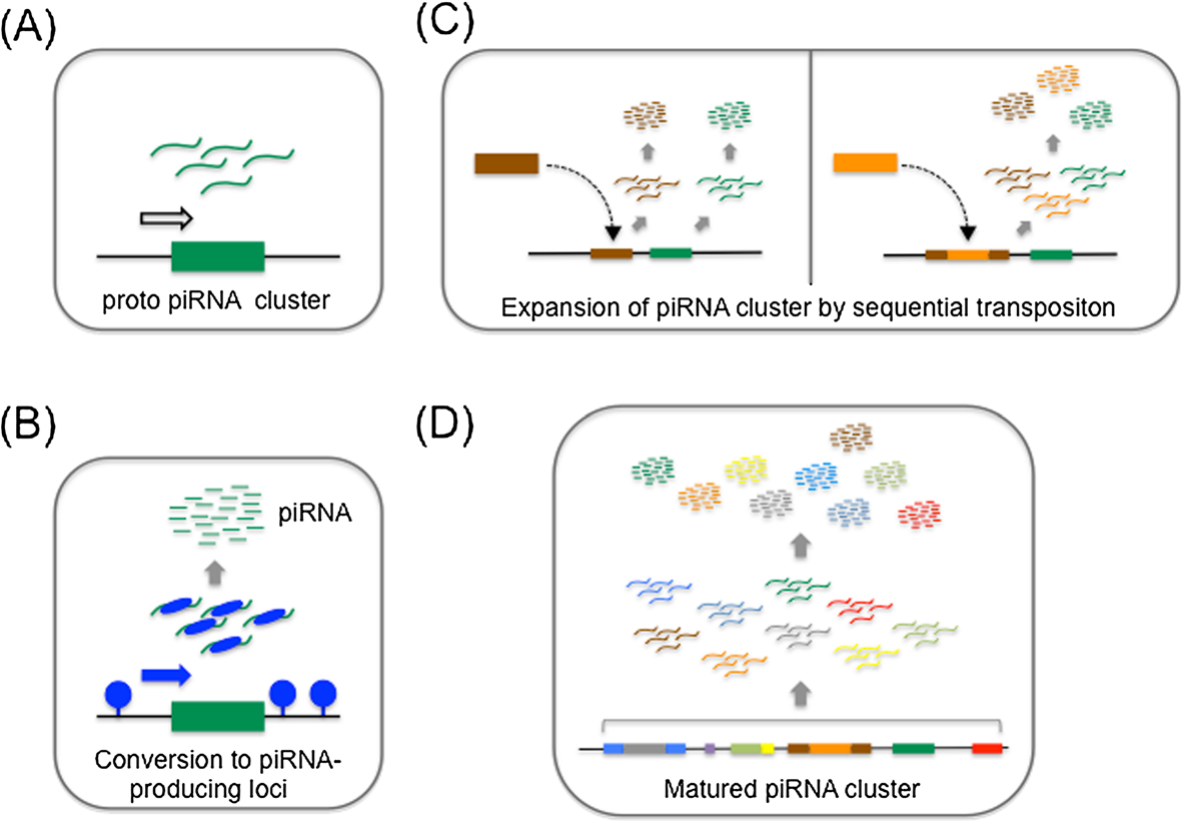
**Model of Piwi-interacting RNA (piRNA) cluster formation. (A)** Proto-piRNA cluster: transcripts are produced from a proto-piRNA-producing locus. **(B)** Conversion to piRNA-producing locus: a specific transcription factor, histone marker, DNA methylation pattern, and/or RNA-binding protein (blue arrow, circle, and oval, respectively) convert the proto-piRNA-producing locus into a piRNA-producing site. **(C)** Sequential transposition event: the open nature of chromatin at the piRNA-producing locus attracts transposon integration (left panel). Certain types of transposons can accept the transposition within themselves (right panel). **(D)** Maturation of piRNA cluster: a mature piRNA cluster is produced through sequential transposition events at piRNA-producing loci.

*Tetrahymena thermophila* has a unique genome processing mechanism, called ‘programmed DNA elimination’. Most ciliated protozoans, including *T. thermophila*, exhibit nuclear dimorphism, with a germline micronucleus (Mic) and somatic macronucleus (Mac) [68]. The genomic sequence of this organism is processed during the course of meiosis. Mic has an unprocessed genome, and Mac has a processed one, but has a much larger genome size due to polyploidy. In contrast to the role of Mic as a reservoir of genetic information, gene expression for maintaining the organism takes place in Mac. The smaller genome size of Mac compared with Mic is attributable to DNA elimination induced by scan RNA (scnRNA). Internal eliminated sequences (IESs) are specific regions that are selectively eliminated from the developing Mac genome, and there are over 6,000 IESs within the Mic genome. scnRNA are loaded onto Twi, one of the *Tetrahymena* PIWI proteins and are, therefore, *T. thermophila* piRNAs [69]. Twi1-scnRNA complexes are then transported to the developing Mac, which has an unprocessed genome, and they recognize and eliminate IESs through base-pairing between IESs and scnRNAs [70]. Strikingly, scnRNA production requires a Dicer-like protein, which is in clear contrast to piRNA production in other animals [71]. scnRNAs map predominantly to IESs, therefore, it can be said that IESs are piRNA clusters in *Tetrahymena*[72]. Recent high throughput analysis has uncovered biased transcription of IESs in Mic; that is, IESs are destined to have high transcription activity [72]. Because of the lack of clear consensus sequence between different IESs, IESs are thought to be epigenetically marked as piRNA clusters.These findings in various animals suggest possible requirements to establish piRNA clusters, which are as follows (in random order): 1) an ability to recruit chromatin-modifying enzymes that contribute to the maintenance of open chromatin so as to attract and trap TEs, 2) an ability to recruit DNA specific factors (for example, specific transcriptional factors) to drive transcription of that region, and 3) an ability to distinguish transcripts from that region from other cellular transcripts and to specifically process them into small RNAs (Figure 6B).

### Transposition and chromatin boundaries

A prerequisite for genomic regions to act as TE traps is that they must be frequent and non-deleterious sites for TE insertion. TEs jump around the genome by transposition, but this appears to occur in a non-random manner [73]. The *P-element* is a DNA transposon that has been used for insertional mutagenesis to isolate specific alleles in *Drosophila*[74,75]. Because of this, a large body of data has accumulated concerning the preferential *P-element* insertion sites in the genome. Analysis of 100,000 transposition events identified that *P-element* insertion preferentially occurs immediately 5′ to genes or within 5′ exons [76]. *piggyBac*, another TE that is also often used for mutagenesis, also shows a high preference of insertion at or near promoter regions of genes [77]. These results indicate that these TEs tend to target genomic regions that presumably contain open chromatin and/or are actively transcribed at the time of transposition.

A fission yeast TE termed *Tf1* is a retrotransposon prevailing in the specific yeast genome. *Tf1* insertion predominantly occurs closer to the 5′ end of genes, in regions known to have relatively open chromatin [78,79]. These studies clearly argue for the relationships between open chromatin and preferential transposition sites. However, it should be noted that these TE insertions at or near promoters alter the transcriptional activity of genes and are, therefore, often highly deleterious to the host. Thus, individual genomes with these insertions tend to be eliminated from the population. So are there any genomic regions where TE insertions are tolerated?

**Figure 7:**
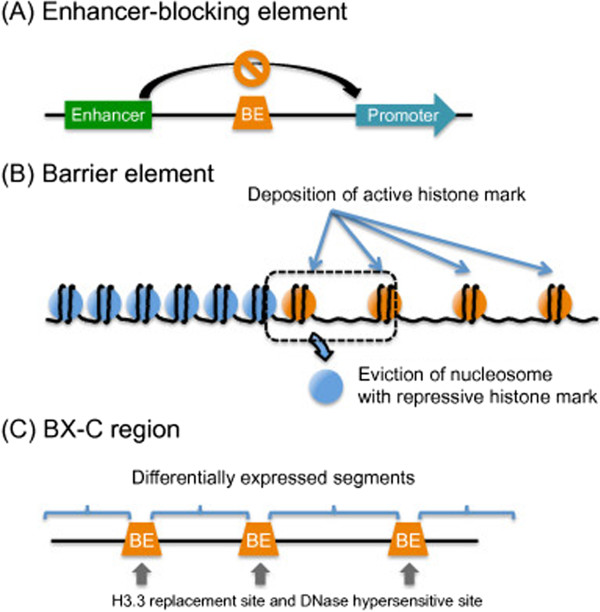
**Three types of boundary elements. (A)** Boundary element intercepts the effect of an enhancer to the nearby promoter. **(B)** Boundary element between heterochromatin and euchromatin serves as a barrier against the spreading of heterochromatin. **(C)** Boundary elements residing in the BX-C region regulate the three homeotic genes to ensure the correct level and pattern of expression, thereby making possible proper segmentation in the *Drosophila* embryo.

In addition to gene promoters and their neighboring regions, chromatin boundaries are also known to have relatively open chromatin structures. A chromatin boundary can act as a buffer between two functional chromatin domains by resisting the proliferation of epigenetic changes that are characteristic of each, thus genes present in one domain are not affected by regulatory sequences present in a different domain [80-84] (Figure 7). *Cis*-regulatory elements are located at chromatin boundaries, and have different compositions of *trans*-acting proteins. They limit the spreading of heterochromatin domains into regions of actively transcribed genes (and *vice versa*) and prevent adventitious interactions between enhancers and promoters when located between them (acting as “insulators”) [83,84] (Figure 7A). However, chromatin boundaries, especially those in *Drosophila*, between constitutive heterochromatin and euchromatin are not fixed but stochastic, as evident in position effect variegation (PEV), in which the heritable inactivating influence of the heterochromatin on a neighboring gene can spread in some, but not all, cells of the same cell type [85].

In fission yeast, tRNA gene clusters near to the site of constitutive heterochromatin, such as those around centromere, serve as strong boundary elements that inhibit the encroachment of heterochromatin into the euchromatic region [86,87] (Figure 7B). One explanation of this phenomenon is that the high transcriptional activity from tRNA genes creates a discontinuity in arrayed nucleosomes that serves as a barrier to the propagation of heterochromatin [88,89]. This high transcriptional activity might also function by promoting the activity of histone-modifying enzymes that contribute to the maintenance of open chromatin conformation [90]. A number of chromatin boundaries are associated with active promoters. In addition, the recruitment of histone acetyltransferase activity correlates well with barrier activity in multiple organisms [82]. These results suggest the possibility that some promoters or transcription units with specific characteristics may determine their own chromosomal environment to ensure their activity, thereby allowing them to effectively resist and even counteract heterochromatin formation, probably by manipulating histone modifications.

In addition to histone modifications, replacement of core histones with their variants appears to contribute to boundary formation. The ENCODE project revealed that specific histone variants are highly abundant at chromatin boundaries. For example, H2A.Z is an evolutionarily conserved H2A variant present in all eukaryotes [91], which exhibits a characteristic localization in genomes, with high concentrations at gene promoters, enhancers, and chromatin boundaries [17,92-95]. These H2A.Z–rich regions are common NDRs, and are therefore DNase-hypersensitive. H2A.Z, together with H3.3, a histone H3 variant, forms histone octamers, which constitute the most labile nucleosome state in human cells. This leads to the dissociation of nucleosomes from chromatin, thereby forming NDRs [93,96]. Mapping the preferential H3.3 deposition sites in *Drosophila* S2 cells revealed that there are specific sites at which H3.3 is heavily deposited [97,98]. The bithorax complex (BX-C) regulates the identity of each of the segments that contributes to the posterior two-thirds of the fly [99]. The region encodes three genes, Ultrabithorax (*Ubx*), abdominal A (*abd-A*), and Abdominal B (*Abd-B)*. It has been shown that nine body segments are defined by the combination of expression level of the three genes. Boundary elements demarcate the BX-C region into nine parts, making possible the differential expression pattern of the three genes. Interestingly, the preferential deposition sites of H3.3 match well with the BX-C boundary elements, such as Fab-7, Fab-8, and Mcp [98]. Moreover, those sites are independently identified as DNase-hypersensitive sites [100] (Figure 7C). Therefore, both H2A.Z and H3.3 serve as molecular indicators of open chromatin conformation. Interestingly, both H2A.Z and H3.3 have been recovered from genome-wide RNAi screening to identify factors required for transposon silencing in *Drosophila*[35]. Thus, it is tempting to speculate that both histone variants are involved in piRNA production, possibly through maintaining the boundary nature of piRNA clusters (see below).

Of note, certain types of TEs themselves also show high rates of H3.3 deposition [97], implying that a TE itself can be a good recipient of a transposon. In addition, it is known that transposition of retrotransposons tends to occur within even older retrotransposons. For example, nearly all retrotransposon insertions in the *Arabidopsis* genome are into older retrotransposons [101,102]. The recent ENCODE project has also revealed that DNase I hypersensitive sites are strongly enriched at specific LTR retrotransposons in the human genome in some cultured cells, suggesting the possibility that TEs can transpose into certain types of TE [95]. This would explain the reason why TEs in piRNA clusters, such as *flamenco*, tend to be arranged in a nested fashion.

Together, these findings suggest that the relatively open nature of chromatin at the chromatin boundary makes this region a susceptible site for TE transposition. We propose a model in which the insertion of a single TE in the chromatin boundary may trigger a runaway process [103]; once the first TE inserts into the region, this site becomes a stretch of landing pads for new TEs, without deleterious consequences. Thus, in effect, any slight concentration of TEs in a chromatin boundary seeds a local TE expansion to produce an even more preferential site or trap for transposition, creating an island or cluster of TEs (Figure 6C, D). It is well known that the *gypsy* retrotransposon serves as an enhancer-blocking insulator, a type of boundary element, when inserted between promoter and enhancer [104]. Therefore, this *gypsy* insulator locus could be a prototype for TE transposition landing pads. The aforementioned findings in *Drosophila*, mouse and other animals also imply that special chromatin status with accompanying transcriptional factors and/or epigenetic factors at the chromatin boundary can give transcriptional license to that region [22,61,66,72]. There is increasing evidence that TEs often carry with them an array of transcription factor binding sites that, when integrated into the genome, can become either alternative promoters or new enhancers [105]. Thus, transposition to a chromatin boundary of a TE that has a specific transcription factor binding site, the transcription factor for which is already expressed in gonads, may make that region transcriptionally active and put it under the control of the transcription factor. In this way, boundary-specific elements may drive transcription of that boundary region to produce transcripts in gonads. A study describing the relationships between TE insertion and *de novo* piRNA production shows that not all TE insertions drive *de novo* piRNA production [106]. The transcriptional status at the insertion site might affect whether the TE transcript is processed into piRNA [106]. This is consistent with the view we have discussed. The chromatin boundaries are gene-poor regions, and therefore TE transposition at those regions is likely to be neutral to the host, thereby allowing not only TE accumulation at those regions, but accumulation of nucleotide changes in those accumulated TEs. Repeated transposition events at the same boundary region would expand the size of clusters. Thus, it is possible that special transcriptional units in the boundary regions are primitive piRNA production sites.

### What makes the piRNA cluster so special?

When thinking about the process by which piRNA clusters are formed, the biggest outstanding question is how does a specific locus turn into a piRNA-producing site? In other words, what is the prerequisite for certain loci to produce piRNAs? We propose two scenarios based on the data described so far.

One model is that piRNA production loci are marked by specific factors. The very recent study from the Theurkauf laboratory revealed that dual-strand transcription and recruitment of Rhino to the corresponding loci trigger piRNA production [107]. Moreover, a study from the Brennecke laboratory showed that Rhino recruits Cutoff, which possibly acts to suppress transcription termination [55]. This implies that Rhino helps Cutoff and other additional factors to recognize nascent transcripts from piRNA clusters, and to distinguish them from other transcripts.

Another model is that transcripts from piRNA clusters have some special property allowing them to be processed into piRNA, and this property is used by the piRNA-producing machinery to distinguish piRNA transcripts from the vast majority of other transcripts. This special property can be either altered splicing, characteristic 3′-end processing, or specific *cis* elements that direct recognition by special *trans* factors. Recently, Madhani and colleagues showed that stalled spliceosomes are a signal for an RNAi response in a human pathogenic yeast, *Cryptococcus neoformans*[108]. These authors proposed that splicing intermediates are a preferred substrate for small interfering RNA biogenesis. This work explains how specific transcripts are differentially recognized by the small RNA biogenesis machinery. It was recently reported that Rhino can suppress normal splicing in the *Drosophila* germ line with the aid of Uap56, making the piRNA precursor transcript different from other pol II transcripts [55,107,109]. However, in *Drosophila* follicle cells, splicing of a long single-stranded transcript (more than 150 kb) produced from the *flam* locus was reported [54]. Furthermore, the first intron of *flam* was found to be constitutively spliced [54]. In addition, there are numerous 3′-end processing signals of TEs located in the *flam* locus. Therefore, there could be a certain mechanism that suppresses transcription termination and poly(A) addition for the *flam* transcripts. Therefore, the transcript itself is sending some message that it is different from other transcript.

## Conclusions

Recent genome-wide ChIP analyses have revealed the locations on the genome where specific transcription and epigenetic factors sit. Cross-linking immunoprecipitation (CLIP) methods have also revealed specific binding sites on transcripts for RNA-binding proteins. There is no doubt that these types of analysis will propel this field forward and expand our knowledge of how piRNA clusters are formed and how transcripts from the clusters are specifically processed into piRNAs. In addition, other methods that are complementary to ChIP and CLIP should also be applied to piRNA research. For example, we do not have a comprehensive understanding of the repertoire of proteins that bind to piRNA clusters or to the transcript from piRNA cluster. Taking advantage of specific DNA-protein interactions, such as LexA with LexA-binding sites, LacI with LacO repeats and modified transcription activator-like effector (TALE), recent studies have successfully immunopurified a chromatin locus of interest and identified associated proteins [110-113]. A combination of RNA-binding proteins and their specific binding sites, such as MS2 and BoxB sites, can be applied to identify the proteins that bind to piRNA transcripts. These types of strategy will allow us to identify the hidden triggers for piRNA production.

## Abbreviations

ChIP: Chromatin immunoprecipitation; CLIP: Cross-linking immunoprecipitation; IES: Internal eliminated sequence; LINE: Long interspersed repetitive element; miRNA: microRNA; NDR: Nucleosome-depleted region; Nt: Nucleotide; PEV: Position effect variegation; piRNA: Piwi-interacting RNA; RISC: RNA-induced silencing complex; scnRNA: Scan RNA; SINE: Short interspersed repetitive element; siRNA: Small interfering RNA; TALE: Transcription activator-like effector; TE: Transposable element; tRNA: Transfer RNA.

## Competing interests

The authors declare that they have no competing interests.

## Authors' contributions

SY and HS designed the structure of the review, and wrote the paper along with MCS. All authors commented on the manuscript, and read and approved the final manuscript.
